# Benzoic acid supplementation in crude protein-deficient diets reduces nursery pig growth performance

**DOI:** 10.1093/jas/skaf279

**Published:** 2025-08-22

**Authors:** Paulo H A de Azevedo, Alex C Outlaw, Lillian Jeffers, Edward T Drabold, Qichen Wang, Brendan T Higgins, Marko Rudar

**Affiliations:** Department of Animal Sciences, Auburn University, Auburn, AL 36849, USA; Department of Animal Sciences, Auburn University, Auburn, AL 36849, USA; Department of Animal Sciences, Auburn University, Auburn, AL 36849, USA; Department of Biosystems Engineering, Auburn University, Auburn, AL 36849, USA; Department of Biosystems Engineering, Auburn University, Auburn, AL 36849, USA; Department of Biosystems Engineering, Auburn University, Auburn, AL 36849, USA; Department of Animal Sciences, Auburn University, Auburn, AL 36849, USA

**Keywords:** antagonistic dietary strategies, glycine, hippuric acid, nitrogen, organic acid, weaned pigs

## Abstract

The objective of this study was to evaluate the interaction between benzoic acid, which is excreted in urine as its Gly conjugate hippuric acid, and low crude protein (CP) diets on nursery pig growth performance. At 28 d age, pigs were weaned and divided into nursery room pens according to body weight and sex (5 mixed-sex pigs per pen). Pigs were fed a commercial starter diet for 4 d; at 32 d age, pigs were weighed (initial body weight, 9.50 ± 0.93 kg) and pens were assigned to one of three dietary treatments: 1) control (CON; 19.8% CP; *n* = 12 pens); 2) low crude protein (LCP; 15.8% CP; *n* = 11 pens) and 3) low crude protein + benzoic acid (LCP + BA; 15.8% CP; 0.9% benzoic acid; *n* = 12 pens). Pigs were fed for 4 wk and pig body weights and feed disappearance were measured weekly to calculate average daily gain (ADG), average daily feed intake (ADFI), and feed efficiency (G:F; gain-to-feed ratio). On days 0, 14, and 28, blood was collected from one pig per pen by jugular venipuncture. Serum was analyzed for hippuric acid and amino acid concentrations. The ADG of CON group (580 ± 11 g/d) was greater than LCP (544 ± 11 g/d) and LCP + BA (503 ± 11 g/d; *P* < 0.05); ADG of LCP was also greater than LCP + BA (*P* < 0.05). There was no difference in ADFI among groups (*P* > 0.05). However, G:F of LCP + BA (0.536 ± 0.006 g/g) was lower than both CON (0.579 ± 0.006 g/g) and LCP (0.576 ± 0.006 g/g; *P* < 0.05); there was no difference in G:F between CON and LCP (*P* > 0.10). Serum hippuric acid concentration was greater in the LCP + BA group compared to either the CON and LCP groups on day 14 and day 28 (*P* < 0.05). Serum Gly concentration in LCP + BA (513 ± 33 µmol/L) was lower than LCP (630 ± 33 µmol/L; *P* < 0.05), whereas Gly of CON (578 ± 33 µmol/L) was intermediate. Serum Lys concentration was lowest in CON (105 ± 9 µmol/L), intermediate in LCP (138 ± 8 µmol/L), and greatest in LCP + BA (167 ± 8 µmol/L; *P* < 0.01). The addition of benzoic acid to CP-deficient diets reduces nursery pig growth performance to a greater extent than CP-deficient diets alone. Greater serum Lys in pigs fed with benzoic acid suggests that Lys was not used as efficiently for growth. Collectively, supplementing benzoic acid in CP-deficient nursery diets could be problematic by decreasing pig growth performance.

## Teaser

Adding benzoic acid to diets deficient in crude protein decreased growth performance in nursery pigs. Since benzoic acid is excreted from pigs in urine as its glycine conjugate, hippuric acid, both glycine and total crude protein content of nursery diets may need to be considered when using benzoic acid as a feed additive.

## Introduction

When feeding nursery pigs, the total supply of crude protein (CP) must meet or exceed the animal requirement for indispensable amino acids (IAA) in addition to providing adequate nitrogen for the endogenous synthesis of dispensable amino acids (DAA; Rocha et al., 2022). Reducing dietary CP, while maintaining sufficient supply of individual IAA and optimal ratios of IAA to Lys, is a sensible goal in swine production systems for three reasons: first, undigested protein entering the large intestine is thought to promote the proliferation of protein-fermenting bacteria that are linked to the development of post-weaning diarrhea (Heo et al., 2009; Pieper et al., 2012; Marchetti et al., 2023), although evidence is inconsistent (Diether et al., 2024; Pearce et al., 2024); second, production and excretion of urea represents an energetic cost to the animal, lowering net energy intake (van Milgen and Dourmad, 2015); and third, increased fecal and urinary nitrogen excretion from excess dietary CP intake can contribute to environmental nitrogen pollution and increased ammonia emissions and other odorous compounds (Trabue et al., 2021). Lowering dietary CP content is accomplished by supplementing crystalline amino acids, mainly Lys, Met, Thr, Trp, and Val (Gloaguen et al., 2014). Rocha et al. (2022) estimated that the minimum dietary CP level and the maximum SID Lys: CP ratio that would not compromise nursery pig average daily gain are 18.4% and 0.066, respectively.

Organic acids have emerged as an alternative to both in-feed antibiotics and zinc oxide to mitigate the negative impacts of post-weaning stress in pigs. In a recent meta-analysis, organic acids improved body weight gain and feed efficiency by approximately 8% and 5%, respectively, but were less effective than in-feed antibiotics (Wang et al., 2022). Among organic acids, benzoic acid is reported to increase growth performance, nutrient digestibility, and gut health parameters in nursery pigs (Kluge et al., 2006; Torrallardona et al., 2007; Diao et al., 2016; Kiarie et al., 2018). Benzoic acid is absorbed rapidly and subsequently conjugated to Gly in the liver, forming hippuric acid, before it is excreted in urine (Kristensen et al., 2009). This is unlike other feed-grade organic acids, such as propionic acid, butyric acid, citric acid, and fumaric acid, which enter anaplerotic or cataplerotic pathways after absorption. While the production and excretion of hippuric acid acidifies urine and reduces ammonia emissions from manure (Humphrey et al., 2022), it also represents an irreversible loss of Gly, and thus nitrogen, from the pig. Pigs can compensate for this loss of Gly by increasing endogenous Gly synthesis using nitrogen derived from other IAA or DAA, but this could become problematic in diets that either approach or are marginally below the estimated protein requirement of the pig, thereby limiting whole-body protein deposition (Wu et al., 2018). Simply put, the combined effect of feeding low CP diets and benzoic acid supplementation on growth performance is unclear. Therefore, the objective of this study was to evaluate the impact of benzoic acid supplementation on growth performance, serum hippuric acid concentration, and serum amino acid concentrations in nursery pigs fed diets deficient in CP. We hypothesized that benzoic acid would exacerbate the reduction in growth performance in pigs fed CP-deficient diets.

## Materials and Methods

All animal procedures were approved by the Institutional Animal Care and Use Committee at Auburn University (PRN 2023-5306).

### Animals and housing

Pigs (Yorkshire × Duroc × Hampshire) were obtained from the Swine Research and Education Center at Auburn University. At 28 d age, a total of 175 pigs were weaned and divided into nursery room pens according to body weight and sex (5 mixed-sex pigs per pen) in three consecutive batches (batch 1, 15 pens; batch 2, 12 pens; batch 3, 9 pens). All pigs were housed in the same nursery room. Pens (1.5 m × 2 m) were equipped with polypropylene flooring positioned above 36-cm deep pits that were flushed weekly for waste management, an adjustable single-sided five-hole nursery feeder (76 cm length × 29 cm width; AP-SN*305; Automated Production Systems, Sterling Heights, MI), and cup waterer (270650; QC Supply, Schuyler, NE). Initial room temperature was set to 29 °C and was reduced by 1.7 °C per week for 3 wk. Pigs were fed a commercial nursery diet for 4 d (metabolizable energy [ME], 3,350 kcal/kg; CP, 21%; standardized ileal digestible [SID] Lys, 1.45%; no in-feed antibiotic or zinc oxide) before starting experimental procedures.

### Animal diets

Three treatment diets were formulated to meet or exceed estimated requirements for all nutrients, except CP for two of three diets, for 10 to 25 kg pigs (NRC, 2012). Diets were formulated based on corn, corn starch, soybean meal, and dried whey (**Table 1**): control (CON; 19.8% CP; 1.26% SID Lys); low crude protein (LCP; 15.8% CP; 1.25%; SID Lys); and low crude protein plus benzoic acid (LCP + BA; 15.8% CP; 1.25% SID Lys; 0.9% benzoic acid). The CP content of the LCP and LCP + BA diets was approximately 15% below estimated requirements for CP (i.e., SID N; NRC, 2012). The targeted total CP content was within 5% of the lower bound of the 95% confidence interval identified by Rocha et al. (2022), ensuring that growth performance would be limited by total CP intake. The amount of benzoic acid added to the LCP + BA diet exceeded the estimated SID Gly content by approximately 20% on a molar basis. Assuming that all benzoic acid is metabolized to hippuric acid, the amount of benzoic acid added was intended to stimulate endogenous Gly synthesis from other DAA and IAA to meet demands for both hippuric acid excretion and growth. Benzoic acid was added to the LCP + BA diet at the expense of corn starch. Diets were collected at mixing and analyzed for crude protein (method 990.03; AOAC Int., 2006) and total amino acids (method 982.30; AOAC Int., 2006) by the University of Missouri Agricultural Experiment Station Chemical Laboratories (**Table 2**). Diets were provided as a mash and fed as a single phase throughout the 28-d period.

**Table 1.** Composition and calculated nutrient content (as-fed basis) of treatment diets

	Diet^1^		
	CON	LCP	LCP + BA
Ingredient, %			
Corn	56.1	40	40
Corn starch	-	22.6	21.7
Soybean oil	2.5	2.5	2.5
Dried whey	10	10	10
Soybean meal, 46.5% CP	28	20	20
L-Lysine•HCl	0.42	0.72	0.72
DL-Methionine	0.15	0.30	0.30
L-Threonine	0.13	0.28	0.28
L-Isoleucine	-	0.05	0.05
L-Tryptophan	-	0.12	0.12
L-Leucine	-	0.19	0.19
L-Valine	0.03	0.24	0.24
L-Histidine•HCl	-	0.13	0.13
L-Phenylalanine	-	0.20	0.20
Limestone	1.10	1.10	1.10
Monocalcium phosphate	0.90	0.90	0.90
Salt	0.45	0.45	0.45
Premix^2^	0.25	0.25	0.25
Benzoic acid^3^	-	-	0.90
Calculated nutrient composition			
ME, kcal/kg	3,415	3,498	3,466
Crude protein, %	19.8	15.8	15.8
SID Lys, %	1.26	1.25	1.25
SID Thr, %	0.76	0.74	0.74
SID Met, %	0.42	0.49	0.49
SID Met + Cys, %	0.69	0.69	0.69
SID Trp, %	0.21	0.21	0.21
SID Val, %	0.81	0.81	0.81
SID Ile, %	0.72	0.65	0.65
SID Gly, %	0.63	0.46	0.46
SID Ser, %	0.81	0.59	0.59
SID Lys: ME, g/Mcal	3.68	3.58	3.62
Gly_equi_,^4^ g/kg	12.1	8.8	8.8
Total Ca, %	0.76	0.73	0.73
Total P, %	0.61	0.52	0.52
STTD P, %	0.38	0.34	0.34
dEB, mEq/kg	234	154	154

^1^CON, control; LCP, low crude protein; LCP + BA, low crude protein plus benzoic acid. Treatment diets were fed for 28 d.

^2^Premix provided the following per kg complete diet: vitamin A, 6600 IU; vitamin D3, 1100 IU; vitamin E, 26 IU; vitamin K, 1.1 mg; vitamin B12, 28 μg; riboflavin, 6.0 mg; niacin, 27.5 mg; and pantothenic acid, 45.1 mg; choline, 110 mg; Fe (as ferrous sulfate), 150 mg; Cu (as copper sulfate), 15 mg; Zn (as zinc sulfate), 150 mg; Mn (as manganous oxide), 40 mg; I (as calcium iodate), 0.5 mg; and Se (as sodium selenite), 0.3 mg.

^3^Benzoic acid was added to the LCP + BA diet such that the ratio of benzoic acid to SID Gly was 1.20 on a molar basis.

^4^Gly equivalent was calculated as the sum of the SID Gly concentration and the molar equivalent of SID Ser; Gly_equi_ (g/kg) = SID Gly (g/kg) + 0.7143 × SID Ser (g/kg).

Abbreviations: dEB, dietary electrolyte balance; ME, metabolizable energy; SID, standardized ileal digestible; STTD, standardized total tract digestible.

**Table 2.** Analyzed crude protein and total amino acid content (as-fed basis) of treatment diets^1^

	Diet^2^		
	CON	LCP	LCP + BA
Crude protein, %	18.9 (19.8)	15.3 (15.8)	14.9 (15.8)
Total Lys, %	1.41 (1.39)	1.33 (1.35)	1.36 (1.35)
Total Thr, %	0.90 (0.88)	0.92 (0.83)	0.75 (0.83)
Total Met, %	0.45 (0.45)	0.47 (0.52)	0.45 (0.52)
Total Val, %	1.00 (0.93)	0.93 (0.90)	0.84 (0.90)
Total Ile, %	0.89 (0.82)	0.75 (0.72)	0.72 (0.72)
Total Leu, %	1.71 (1.66)	1.45 (1.41)	1.35 (1.41)
Total Phe, %	0.97 (0.93)	0.88 (0.87)	0.85 (0.87)
Total His, %	0.51 (0.51)	0.47 (0.48)	0.44 (0.48)
Total Gly, %	0.78 (0.75)	0.56 (0.54)	0.53 (0.54)
Total Ser, %	0.82 (0.93)	0.60 (0.68)	0.59 (0.68)

^1^Values in parentheses indicate formulated values (NRC, 2012).

^2^CON, control (19.8% CP; 1.25% SID Lys); LCP, low crude protein (15.8% CP; 1.25% SID Lys); LCP + BA, low crude protein plus benzoic acid (15.8% CP; 1.25% SID Lys; benzoic acid, 0.9%). Treatment diets were fed for 28 d.

### Experimental procedures and sample collection

At 32 d age, pigs were weighed (initial body weight, 9.50 ± 0.93 kg) and pens were assigned to one of three dietary treatments: 1) CON (*n* = 12 pens); 2) LCP (*n* = 11 pens); and 3) LCP + BA (*n* = 12 pens). Pigs were fed for 28 d; feed addition per pen was weighed and recorded daily, and pig body weight and pen feed disappearance were measured weekly to calculate average daily gain (ADG), average daily feed intake (ADFI), and feed efficiency (G:F; gain-to-feed ratio). Feed and water access were provided ad libitum. Pig morbidity, mortality, and removals were monitored daily. On days 0, 14, and 28, feed was removed from each pen feeder with a wet/dry vacuum. After withholding access to feed for 2 h, the pig closest to the mean body weight of the pen was selected and bled by jugular venipuncture. Blood (10 mL) was collected into serum vacutainer tubes (BD, Franklin Lakes, NJ) and allowed to clot at room temperature. Serum was subsequently separated from whole blood by centrifugation (3,000 × g for 20 min at room temperature), aliquoted, and stored at −20 °C until further analysis.

### Sample processing and laboratory analysis.

#### Serum hippuric acid

Serum was filtered through 0.2-µm nylon membranes before analysis by high-performance liquid chromatography (HPLC). The HPLC system (Prominence, Shimadzu, Japan) consisted of a controller (CBM-20A), two pumps (LC-20AD), an autosampler (SIL-20AC), a column oven (CTO-20A), and a diode array detector (SPD-M20A). Hippuric acid was separated on an analytical column (Acclaim Polar Advantage II column, 3 µm, 3 mm × 150 mm; Thermo Fisher Scientific, Waltham, MA). Mobile phases were 1 mg/mL ammonium acetate and 5% v/v methanol (A) and methanol (B). Separation of hippuric acid was performed at a flow rate of 0.5 mL/min with the following linear gradient: 0 min, 0% B; 0-12 min, 40% B; 12-16 min, 40% B; 16-18 min, 0% B; and 18-20 min, 0% B. The autosampler and column oven temperatures were set at 4 °C and 50 °C, respectively. The sample injection volume was 20 µL. Peaks were monitored at 220 nm.

An external calibration curve was constructed from eight concentrations of sodium hippurate standard (0 to 250 mg/L; two-fold serial dilution), corresponding to 0 to 223 mg/L hippuric acid. Hippuric acid standards were analyzed by HPLC as described above. Linear regression analysis of standard hippuric acid concentrations (*R*^2^ = 1) and peak areas was used to calculate sample hippuric acid concentrations.

#### Serum amino acids

Serum amino acid concentrations were analyzed by ultra-high performance liquid chromatography (UHPLC) with fluorescence detection following sample deproteinization and derivatization with 6-aminoquinolyl-N-hydroxysuccinimidyl carbamate (AQC), as described previously (Outlaw et al., 2023). Serum Cys and homocysteine concentrations were analyzed by UHPLC with fluorescence detection following sample reduction, deproteinization, and derivatization with 4-fluoro-7-aminosulfonylbenzofurazan (ABD-F), as described previously (Gachman et al., 2024).

#### Serum urea nitrogen

Serum urea nitrogen concentration was measured enzymatically using a commercial kit from kinetic analysis (Urea Nitrogen (BUN) Liqui-UV (Rate); Stanbio Laboratory, Boerne, TX). Absorbance was quantified with a Synergy 4 multimode plate reader (BioTek Instruments, Winooski, VT).

#### Calculations and statistical analysis

Data were analyzed using the generalized linear mixed model procedure of SAS (version 9.4; SAS Institute, Inc., Cary, NC). Growth performance (initial body weight, final body weight, average daily gain, average daily feed intake, and gain-to-feed ratio) was analyzed by one-factor ANOVA. The statistical model included dietary treatment as a fixed effect and pen and batch as random effects; initial body weight was included as a covariate for final body weight. Pen was considered the experimental unit. Serum hippuric acid, amino acid, and urea concentrations were analyzed by two-factor ANOVA. The statistical model included dietary treatment, time, and their interaction as fixed effects and pig and batch as random effects. Pig was considered the experimental unit; since the same pig per pen was not sampled on days 0, 14, and 28, the pig was not considered a repeated measure for this analysis. For serum hippuric acid only, a lognormal transformation was applied to normalize the distribution of residuals; results were back transformed for data presentation. For all analyses, a variance component structure was used to model random effect variance, and degrees of freedom were estimated with the Satterthwaite method. Normality of residuals was assessed with Shapiro-Wilk test statistics. Orthogonal contrasts were constructed to test the difference in serum hippuric acid, serum Gly, and serum urea concentrations between the LCP and LCP + BA groups on days 0, 14, and 28. Data are presented as least-squares means ± standard error of the mean; differences among least-squares means were considered significant at *P *≤ 0.05 and a trend at 0.05 < *P* ≤ 0.10.

## Results

Amino acid analysis of treatment diets was largely consistent with their calculated nutrient contents (**Table 2**). The calculated Gly_equi_ (total basis), which was calculated as the sum of the total Gly content (g/kg) and the molar equivalent of total Ser content (g/kg), in the LCP (9.9 g/kg) and LCP + BA (9.5 g/kg) diets were 28% and 30% lower, respectively, than the CON diet (13.7 g/kg). This relative reduction in Gly_equi_ was consistent when it was also calculated on an SID basis. One pen from the LCP group was removed from analysis due to low feed intake (~30% less than other treatments) resulting from incorrect feeder adjustment. Pig removal and mortality rates were minimal and not different among treatments.

### Growth performance

Initial body weight did not differ among treatment groups (**Table 3**; *P* > 0.10). Over the 28-d experiment, ADG was lower in the LCP + BA group compared to both the CON and LCP groups, and ADG was lower in the LCP group compared to the CON group (*P* < 0.01). While ADFI tended to be lower in the LCP and LCP + BA groups compared to the CON group (*P* = 0.06), ADFI was not different between the LCP and LCP + BA groups (*P* > 0.10). Accordingly, G:F was not different between the CON and LCP groups (*P* > 0.10), whereas G:F was lower in the LCP + BA group compared to the CON and LCP groups (*P* < 0.01). Overall, final body weight of the LCP + BA group was lower than both the CON and LCP groups, and the final body weight of the LCP group was lower than the CON group (*P* < 0.01).

**Table 3.** Effect of dietary treatment on starter pig growth performance

	Diet^1^				
Item	CON	LCP	LCP + BA	SEM^2^	*P*-value
Initial BW, kg	9.50	9.48	9.53	0.29	0.99
Final BW, kg	25.75^a^	24.74^b^	23.58^c^	0.29	<0.01
ADG, g/d	580^a^	544^b^	503^c^	11	<0.01
ADFI, g/d	1001	944	941	20	0.06
G:F, g/g	0.579^a^	0.576^a^	0.536^b^	0.006	<0.01

^1^CON, control (19.8% CP; 1.25% SID Lys); LCP, low crude protein (15.8% CP; 1.25% SID Lys); LCP + BA, low crude protein plus benzoic acid (15.8% CP; 1.25% SID Lys; benzoic acid, 0.9%). Treatment diets were fed for 28 d.

^2^Maximum value for the standard error of the mean.

^a,b^Means without a common superscript differ after Tukey multiple comparison test, *P* < 0.05.

Abbreviations: CP, crude protein; G:F, gain-to-feed ratio; SID, standardized ileal digestible.

### Serum hippuric acid

**Figure 1.**
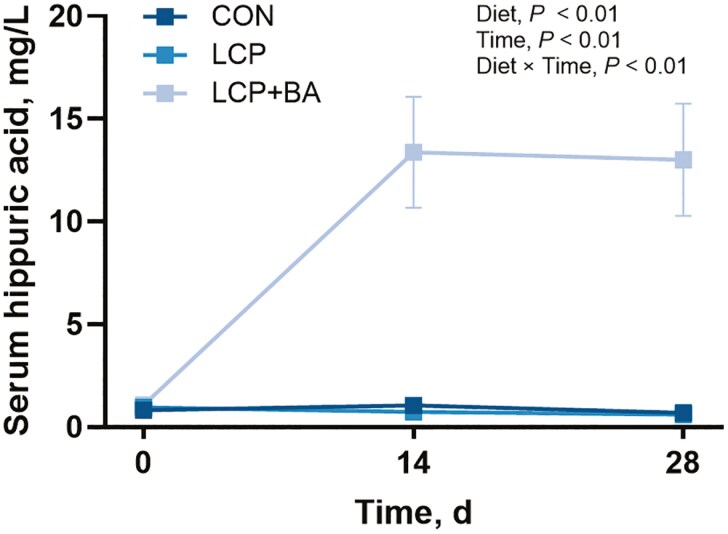
Serum hippuric acid concentrations (mg/L) in nursery pigs fed control (CON; 19.8% CP; *n* = 12), low protein (LCP; 15.8% CP; *n* = 11), or low protein with benzoic acid (LCP + BA; 15.8% CP, 0.9% benzoic acid; *n* = 12) diets for 28 d. Values are least-squares means ± standard error of the mean. The maximum standard error of the mean for the CON and LCP data points is 0.215; the error bars are not visible due to symbol size.

Serum hippuric acid concentration was not different among groups on day 0 (*P* > 0.10). Hippuric acid was greater in the LCP + BA group compared to either the CON or LCP groups on day 14 (LCP + BA, 13.38 mg/L; CON, 1.06 mg/L; LCP, 0.74 mg/L) and on day 28 (LCP + BA, 13.01 mg/L; CON, 0.69 mg/L; LCP, 0.61 mg/L) (*P* < 0.01; **Figure 1**). Serum hippuric acid was not different between the CON and LCP groups on any day (*P* > 0.10).

### Serum amino acids

Among DAA, Gly was the only amino acid that was lower in the LCP + BA group compared to the LCP group (*P* < 0.05; **Table 4**); all other DAA, including Ser, were not different between the LCP and LCP + BA groups (*P* > 0.10). This was especially evident on day 14 when serum Gly concentration was 39% lower in the LCP + BA group compared to the LCP group (*P *< 0.01), whereas it was only 17% lower on day 28 (*P *= 0.05). Total DAA, however, was not different among groups (*P* > 0.10).

**Table 4.** Effect of dietary treatment, day, and their interaction on serum amino acid concentrations (µmol/L)

Day	0			14			28				*P*-value		
Diet^1^	CON	LCP	LCP + BA	CON	LCP	LCP + BA	CON	LCP	LCP + BA	SEM^2^	Diet	Day	Diet × Day
Amino acid^3^													
Ala	384	383	426	320	390	409	214	268	318	27	<0.01	<0.01	0.56
Arg	101	104	84	110	92	105	95	84	93	7	0.11	0.05	0.04
Asn	36	36	31	66	60	62	50	49	56	5	0.82	<0.01	0.60
Asp	11	12	9	11	11	10	10	9	10	1	0.35	0.51	0.27
Cit	32	35	34	37	36	31	31	30	32	3	0.77	0.15	0.30
Cys	153	150	149	129	135	135	136	134	136	6	0.98	<0.01	0.73
Glu	109	104	90	91	100	94	72	82	91	9	0.79	0.02	0.30
Gln	293	299	305	414	415	349	322	369	391	21	0.45	<0.01	<0.01
Gly	582	604	625	611	707	433	542	577	481	44	<0.01	0.04	<0.01
Hcy	27	22	23	39	43	34	52	66	57	4	0.24	<0.01	0.25
His	33	33	33	28	34	45	34	41	48	5	<0.01	<0.01	0.04
Ile	69	65	65	72	68	82	68	62	72	4	0.07	0.03	0.38
Leu	85	82	70	114	110	123	115	108	116	7	0.57	<0.01	0.11
Lys	100	113	89	107	167	235	108	133	177	13	<0.01	<0.01	<0.01
Met	33	40	36	25	62	93	22	47	51	7	<0.01	<0.01	<0.01
Orn	44	48	42	72	64	66	64	55	64	5	0.54	<0.01	0.40
Phe	51	51	47	46	49	56	46	48	50	3	0.16	0.33	0.06
Pro	147	147	148	176	186	179	156	152	157	14	0.96	<0.01	0.95
Ser	73	75	69	116	116	104	115	104	105	9	0.25	<0.01	0.80
Tau	58	63	51	30	76	65	54	73	75	7	<0.01	<0.01	<0.01
Thr	102	126	108	146	261	346	133	188	254	19	<0.01	<0.01	<0.01
Tyr	74	89	81	124	102	99	112	95	91	9	0.02	<0.01	<0.01
Val	88	88	73	108	189	222	138	181	207	9	<0.01	<0.01	<0.01
Total BCAA	241	235	208	294	365	427	321	351	395	19	<0.01	<0.01	<0.01
Total IAA	662	705	607	757	1032	1308	760	894	1069	56	<0.01	<0.01	<0.01
Total DAA	1863	1902	1933	2058	2223	1874	1730	1840	1835	93	0.17	<0.01	0.09

^1^CON, control (19.8% CP; 1.25% SID Lys); LCP, low crude protein (15.8% CP; 1.25% SID Lys); LCP + BA, low crude protein plus benzoic acid (15.8% CP; 1.25% SID Lys; benzoic acid, 0.9%). Treatment diets were fed for 28 d.

^2^Maximum value for the standard error of the mean.

^3^Tryptophan concentrations were not measured because the tryptophan-AQC derivative is not detectable by fluorescence.

Abbreviations: BCAA, branched-chain amino acids; Cit, citrulline; DAA, dispensable amino acids; Hcy, homocysteine; IAA, indispensable amino acids; Orn, ornithine; Tau, taurine.

Conversely, total IAA were greater in the LCP + BA group compared to the CON and LCP groups and total IAA were greater in the LCP group compared to the CON group (*P* < 0.01; **Table 4**). Individual IAA, including Lys, Thr, His, Val, in addition to total branched-chain amino acids (BCAA), paralleled treatment differences in total IAA. In contrast to serum Gly, serum Lys concentrations were 41% greater in the LCP + BA group compared to the LCP group on day 14 and 33% greater on day 28.

### Serum urea concentration

**Figure 2.**
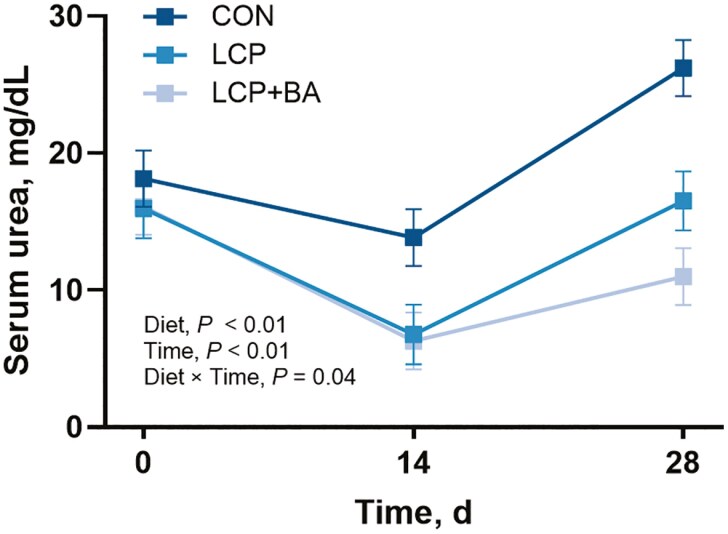
Serum urea concentrations (mg/dL) in nursery pigs fed control (CON; 19.8% CP; *n* = 12), low protein (LCP; 15.8% CP; *n* = 11), or low protein with benzoic acid (LCP + BA; 15.8% CP, 0.9% benzoic acid; *n* = 12) diets for 28 d. Values are least-squares means ± standard error of the mean.

Serum urea concentration was not different among groups on day 0 (*P* > 0.10). Serum urea was greater in the CON group compared to either the LCP or LCP + BA groups on day 14 and on day 28 (*P* < 0.01; **Figure 2**). Serum urea was not different between the LCP and LCP + BA groups on day 0 or on day 14 (*P* > 0.10) but tended to be lower in the LCP + BA group on day 28 (*P* = 0.07).

## Discussion

Low CP diets and benzoic acid supplementation are two separate approaches used in nursery pig diet formulation. Low CP diets minimize total protein intake, whereas benzoic acid promotes gastric acidification and protein digestibility. Both approaches ostensibly work to reduce the flow of undigested protein to the hindgut and limit the proliferation of protein-fermenting bacteria thought to be associated with post-weaning diarrhea in nursery pigs (Heo et al., 2009; Pieper et al., 2012; Marchetti et al., 2023). Since benzoic acid is first conjugated to Gly in the liver before the product, hippuric acid, is excreted in urine, and dietary benzoic acid is almost entirely recovered in urinary hippuric acid (Kristensen et al., 2009), an antagonism between feeding low CP diets and benzoic acid supplementation is possible, wherein nitrogen is lost irreversibly from pigs as hippuric acid. This, in turn, could limit total DAA availability for whole-body protein deposition and growth. Therefore, we investigated the interaction between feeding CP-deficient diets and benzoic acid supplementation on nursery pig growth performance.

Both the LCP and LCP + BA groups exhibited lower body weight gain and final body weights compared to the CON group. While previous studies have shown that low CP diets supplemented with crystalline amino acids do not adversely affect pig growth performance (Heo et al., 2008; Morales et al., 2015; Zhou et al., 2016; Duarte et al., 2024; Pearce et al., 2024), other studies have reported the opposite effect (Nyachoti et al., 2006; Opapeju et al., 2008; He et al., 2016; Wu et al., 2018; Yu et al., 2019). The discrepancy across studies, including the present study, could be attributed to the amino acid supplementation strategy. For example, low protein nursery diets (e.g., ~17% CP) must be supplemented with crystalline Ile and Val, in addition to Lys, Met, Thr, and Trp; very low protein nursery diets (e.g., ~14%) will additionally need supplemental crystalline Leu, His, and Phe, none of which are routinely used in conventional diet formulations, to meet estimated IAA requirements (Rocha et al., 2022). When CP level is below 18.4% (Rocha et al., 2022), nitrogen itself could become limiting for DAA production, in turn reducing whole-body protein deposition and overall growth performance (Gloaguen et al., 2014; Mansilla et al., 2017; Wu et al., 2018). In the current study, the LCP and LCP + BA diets supplied all IAA, including Leu, His, and Phe, but pigs fed these diets still demonstrated reduced growth performance compared to the CON diet. The total CP level and SID Lys: CP ratio in the LCP and LCP + BA diets was ~18% lower and ~20% greater, respectively, than the estimated breakpoints for maximum growth performance in nursery pigs (Rocha et al., 2022). A component of the observed reduction in growth performance could also be due to the trend for lower ADFI, perhaps caused by differences in dietary electrolyte balance (dEB), in the LCP and LCP + BA groups compared to the CON group. The dEB in the LCP and LCP + BA diets (154 mEq/kg) was ~34% lower than the CON diet (234 mEq/kg). Although the dEB in all diets fell within the range in which optimal growth can occur (0–600 mEq/kg; NRC, 2012), Jones et al. (2019) reported that ADFI decreases linearly with decreasing dEB from 248 to 84 mEq/kg (phase 1 nursery diet) and 199 to 29 mEq/kg (phase 2 nursery diet). However, the reduction in growth performance in LCP + BA compared to LCP cannot be attributed to differences in dEB or in ADFI.

In the current study, the measured concentration of serum hippuric acid in the LCP + BA group on day 14 and on day 28 were comparable but marginally lower than that reported by Kristensen et al. (2009) who meal-fed growing pigs diets top-dressed with 10 g/kg benzoic acid. The measured concentration of serum Gly in the LCP compared to the CON group is consistent with Figueroa et al. (2003), who reported increased Gly levels in growing pigs fed diets with 11% CP compared to 16% CP. Conversely, Gly in the LCP + BA group was lower than that of the LCP group. The discrepancy in serum Gly concentrations between the LCP and LCP + BA group likely reflects substantial Gly partitioning toward hippuric acid production in the LCP + BA group. This finding is also consistent with lower growth performance in the LCP + BA group compared to the LCP group. Increased use of Gly for hippuric acid production likely reduced the availability of Gly (and nitrogen) for protein deposition because nitrogen, derived from other DAA and IAA, was needed to produce Gly. Glycine can be synthesized in pigs from several amino acid precursors including Ser via serine hydroxymethyltransferase, Thr via threonine dehydrogenase, and 4-hydroxyproline via hydroxyproline oxidase and alanine-glyoxylate amino transferase (Wang et al., 2013). The lack of difference in serum Ser, Ala, and other DAA between the LCP and LCP + BA groups, and the increase in serum Thr in the LCP + BA group, suggests that these amino acids were not disproportionately used for endogenous Gly synthesis. Concentrations of Ser, Ala, and Thr, however, are not necessarily indicative of shifts in their flux. While this assertion could also be made for Gly, the increase in serum hippuric acid, an irreversible end-product of Gly metabolism, and corresponding reduction in serum Gly in the LCP + BA group together imply increased Gly utilization toward hippuric acid production and, accordingly, away from whole-body protein deposition. It is possible that pigs did not increase endogenous Gly synthesis enough to offset the greater Gly demand for benzoic acid excretion (Powell et al., 2011; Wang et al., 2014). It is also possible that increased Gly demand indirectly impaired skeletal muscle growth through diminished Gly-mediated activation of the mechanistic target of rapamycin signaling pathway (Sun et al., 2016; Caldow et al., 2019).

Despite the LCP and LCP + BA diets providing the same amount of CP and IAA, the LCP + BA group had greater serum concentrations of Lys, Thr, and total IAA (paired with lower growth performance, as discussed above) compared to the LCP group. Accounting for the CON group also, there was an overall trend for an inverse relationship between serum IAA concentrations and growth performance. This suggests that IAA in the LCP and LCP + BA groups was used inefficiently for whole-body protein deposition compared to the CON group, even though SID Lys and SID Thr content among diets was similar. Feeding benzoic acid appeared to exacerbate this inefficiency, underscoring that benzoic acid depleted nitrogen that otherwise would have been available for growth. Although the trend for lower serum urea concentration in the LCP + BA compared to the LCP group on day 28 implies improved nitrogen retention, we speculate that nitrogen that is intended for excretion as urea was instead excreted as hippuric acid (containing nitrogen derived from Gly) in pigs fed the LCP + BA diets. The observed pattern in amino acid concentrations is consistent with Figueroa et al. (2003) and Morales et al. (2015), who reported that plasma Lys concentrations increased when dietary CP was reduced in growing pigs. However, we cannot rule out the possibility that greater serum IAA concentrations in the LCP and LCP + BA groups were, at least in part, a product of increased dietary inclusion of crystalline amino acids that are absorbed rapidly compared to protein-bound amino acids that require digestion before absorption (Morales et al., 2015). Lastly, in the LCP + BA group, total IAA and Lys concentrations peaked on day 14 and coincided with minimum Gly concentrations, indicating that amino acids were utilized less efficiently on day 14 than on day 28. On day 28, however, the reduction in relative amino acid and total CP requirements (as a percent of diet) appeared to sufficiently compensate for the corresponding increase in benzoic acid intake as total feed intake increased, such that serum Lys, total IAA, and Gly concentrations were not as different in the LCP + BA group compared to the LCP group.

The reduction in nursery pig growth performance observed in pigs fed CP-deficient diets supplemented with benzoic acid contrasts with previous findings in pigs fed conventional diets, where benzoic acid supplementation has been associated with improved growth performance. While some studies show either no or minimal improvements in nursery pig growth performance with added benzoic acid (Choi et al., 2023; Outlaw et al., 2023), the consensus is that benzoic acid inclusion, typically added to nursery diets at 5 g/kg and optimally at 6 g/kg, increases ADG, ADFI, and G:F by 9.5%, 6.1%, and 3.2%, respectively, as reviewed recently by Choi and Kim (2024). Importantly, however, the observed increases in growth performance were in nursery pigs fed diets that were not deficient in CP. Thus, the positive effect of benzoic acid on nursery pig growth performance could be attributed to counteracting total diet acid binding capacity-4, reducing digesta pH, increasing protein digestibility, and minimizing enteric pathogen proliferation (Choi and Kim, 2024), whereas the negative effect of benzoic acid on growth performance in the current study could be attributed to a further reduction in total nitrogen available for growth.

## Conclusion

In summary, the current study confirmed our hypothesis that the addition of benzoic acid to CP-deficient diets decreases growth performance in nursery pigs. Since hippuric acid production requires Gly, our results point toward limited capacity of the nursery pig to maintain endogenous Gly synthesis that supports both benzoic acid excretion and body weight gain when fed CP-deficient. Accordingly, both Gly and CP content of nursery diets may need to be considered when using benzoic acid as a feed additive if the CP level is low. Future work will investigate the impact of increasing benzoic acid supplementation in CP-deficient diets on nitrogen balance and whole-body protein metabolism and the effect of benzoic acid supplementation on growth performance, protein digestibility, gut health, and diarrhea status in nursery pigs fed conventional nursery diets that meet, but not exceed, the estimated CP requirement of the pig.

## Abbreviations

ABD-F: 4-aminosulfonyl-7-fluoro-2,1,3-benzoxadiazole
AQC: 6-aminoquinolyl-N-hydroxysuccinimidyl carbamate
dEB: dietary electrolyte balance
CP: crude protein
DAA: dispensable amino acid
IAA: indispensable amino acid
SID: standardized ileal digestible.
